# Event-Triggered Fixed-Time Integral Sliding Mode Control for Nonlinear Multi-Agent Systems with Disturbances

**DOI:** 10.3390/e23111412

**Published:** 2021-10-27

**Authors:** Xue Li, Zhiyong Yu, Haijun Jiang

**Affiliations:** College of Mathematics and System Sciences, Xinjiang University, Urumqi 830046, China; lixuejiayouya@163.com (X.L.); jianghai@xju.edu.cn (H.J.)

**Keywords:** multi-agent systems, sliding mode control, leader-following consensus, fixed-time

## Abstract

In this paper, the leader-following consensus problem of first-order nonlinear multi-agent systems (FONMASs) with external disturbances is studied. Firstly, a novel distributed fixed-time sliding mode manifold is designed and a new static event-triggered protocol over general directed graph is proposed which can well suppress the external disturbances and make the FONMASs achieve leader-following consensus in fixed-time. Based on fixed-time stability theory and inequality technique, the conditions to be satisfied by the control parameters are obtained and the Zeno behavior can be avoided. In addition, we improve the proposed protocol and propose a new event-triggering strategy for the FONMASs with multiple leaders. The systems can reach the sliding mode surface and achieve containment control in fixed-time if the control parameters are designed carefully. Finally, several numerical simulations are given to show the effectiveness of the proposed protocols.

## 1. Introduction

In the past several years, more and more researchers are interested in cooperative control of multi-agent systems (MASs) because of its robustness, flexible deployment and high efficiency. Cooperative control is widely used in various research fields to solve engineering and non-engineering problems, such as formation of robots [1], sensor networks [2], attitude alignment [3] and so on. Among multitudinous cooperative control objectives, consensus is a basic problem in MASs. Its purpose is to design a controller which can ensure that all members agree on an interest signal according to local information. Therefore, the information exchange between agents on the shared network is regulated by the consensus algorithm or protocol.

Based on observation of nature, the emergence of leaders in animal groups led to the development of the leader-following problem in collective behavior of MASs. In the distributed consensus problem, the existing results of MASs can be roughly divided into three categories according to the number of leaders: leaderless consensus [4,5,6], leader-following consensus [7,8,9] and containment control of multiple leaders [10,11]. In [4], the leaderless consensus of discrete-time MASs was studied by considering the connectivity of the network. In [5], the leaderless consensus of model-independent MASs was considered. In [6], the leaderless consensus of fractional-order MASs was investigated. In the case of single leader, the leader-following bipartite consensus problem was investigated for linear MASs in [7]. The leader-following consensus for MASs with Lipshitz-type node dynamics was considered in [8]. Furthermore, by using distributed impulsive control method, the authors studied the leader-following consensus of nonlinear MASs in [9]. In the case of multiple leaders, the reduplicative learning control problem for nonlinear heterogeneous MASs was investigated in [10]. In [11], a completely distributed control protocol was proposed to study the time-varying group formation tracking problem for linear MASs with multiple leaders.

In the consensus analysis of MASs, the convergence rate is an important index to evaluate the effectiveness of the proposed protocol. Most of existing results mainly concerned with the asymptotic convergence of the system. Due to the rapid development of finite-time theory, some researchers developed the finite-time consensus protocols [12,13,14]. In [12], the authors investigated the practical finite-time consensus of second-order heterogeneous switched nonlinear MASs. In [13], the authors investigated the distributed finite-time tracking control problem for second-order MASs, and proposed a novel observer-based control algorithm. In [14], the finite-time control law for continuous FONMAS was proposed, which ensures that the obstacles in the way can be passed by all agents, and the relative position between two agents reaches a constant value in finite-time. In proposed finite-time protocols, the estimation of convergence time depends on the initial values of MASs. To overcome this shortcoming, the researchers developed the fixed-time consensus protocols. In [15], the fixed-time leader-following flocking for second-order MASs was studied. For fixed-time consensus of heterogeneous MASs, the protocol based on neighbors’ states was proposed in [16], and the state observation control protocol was designed in [17].

Compared with the traditional continuous control, the sampling control can effectively reduce the communication update frequency, so as to reduce the control cost. Therefore, the sampled-data control method was applied to study the issue of resilient reliable dissipativity performance index for systems including actuator faults and probabilistic time-delay signals in [18]. However, the traditional sampled-data control method is time-dependent, which requires the controller to be updated regularly even if the control goal is achieved. This method also leads to some unnecessary waste of computational and communication resources. The event-triggered protocol is used as an efficient method to further reduce communication and computing load. Therefore, the event-triggered controller was designed to study the consensus of first-order MASs in [19]. The consensus of linear MASs was studied via observer-based event-triggered control and two novel schemes were proposed in [20]. The containment control of second-order nonlinear MASs was considered based on event-triggered method in [21]. The consensus problem for a kind of stochastic MASs was studied and an adaptive output feedback approach based on event-triggered was proposed in [22]. To our knowledge, there is little research on the fixed-time consensus of MASs under event-triggered control protocol.

Most of the work mentioned above considers the ideal environment, but agents may face various disturbances signals or noise in communication. As pointed out in [23], disturbances signals or noise can destroy some good properties of a system. Therefore, the consensus of MASs under imperfect environment is worth considering. In [24], the consensus problem for linear MASs with the heterogeneous disturbances generated by the Brown motion was investigated. In [25], the authors investigated the distributed finite-time optimization issue for second-order MASs with matched interferences. By using disturbance rejection strategy, the event-triggered output consensus for MASs with time-varying disturbances was considered in [26]. Furthermore, the fixed-time event-triggered consensus for high-order and second-order MASs with uncertain disturbances was studied in [27,28], respectively.

Since the sliding mode technology can achieve a fast convergence rate to suppress disturbances, sliding mode control (SMC) method is widely used in the control of MASs with disturbances. In [29], a sliding mode estimator was given to accomplish distributed consensus for MASs. The sliding mode controllers were proposed for second-order MASs with mismatched uncertainties in [30]. The adaptive SMC protocols were designed to study the consensus of MASs with unknown disturbances in [31]. In order to improve the convergence rate, the finite-time SMC protocol was proposed in [32]. Furthermore, by using integral terminal SMC, the fixed-time consensus tracking issue for second-order MASs was investigated under the influence of interference signals in [33]. In these works [29,30,31,32,33], all control protocols were continuously updated. In order to reduce the control costs, considering the external interference, the event-triggered integral SMC was proposed to study the time-varying formation control of high-order MASs in [34]. Moreover, the event-triggered finite-time consensus for multirobot systems with disturbances was considered via integral SMC strategy in [35]. The finite-time consensus for nonholonomic MASs with disturbances was studied by using event-triggered integral SMC method in [36]. However, in the existing research, the fixed-time event-triggered integral SMC method for single leader and multiple leaders of MASs with disturbances are rarely considered.

Inspired by the above considerations, this paper studies the fixed-time consensus problem for FONMASs with single leader and multiple leaders by using integral SMC and the theory of fixed-time stability. Firstly, to study the fixed-time leader-following consensus of FONMASs with external disturbances, a new event-triggered integral SMC protocol is devised for each agent. We show that both the systems can get to the sliding mode surface and all agents will achieve consensus in fixed-time under the proposed protocol. Moreover, the FONMAS with multiple leaders and external disturbances is considered. By generalizing our proposed event-triggered integral SMC protocol, it is proved that the containment control can also be achieved and the disturbance can be effectively suppressed in fixed-time. Compared with the existing works, the main contributions of the paper are at least the following three points:

1. In existing works [27,28], the disturbance rejection method was applied to study the fixed-time consensus of MASs with external disturbances. However, the integral SMC method combination with event-triggered control mechanism are introduced to design the distributed protocol in this paper, which can effectively suppress the disturbances and achieve consensus in fixed-time.
2. In [35,36], the finite-time event-triggered integral SMC protocols were proposed, in which the estimation of settling time was associated with the initial conditions. To overcome this disadvantage, the fixed-time event-triggered integral SMC protocols are proposed in this paper. According to the stability theory of fixed-time, we can prove that the consensus can be reached in fixed-time and the upper bound estimation of settling time is regardless of the initial conditions of MASs.
3. The containment control for FONMASs with multiple leaders is also considered, in which a generalized event-triggered integral SMC protocol is designed and the controller is updated only at some discrete instants. The sliding surface and the containment control can be reached in fixed-time. The Zeno phenomenon can be avoided.

The remainder of this paper is organized as follows. Section 2 introduces some preliminaries including graph theory, definitions, lemmas and problem formulation. In Section 3, the consensus protocols based on SMC technique are proposed, and some theorems are proved. In Section 4, the effectiveness of the proposed control protocols is verified by numerical simulations. Some conclusions are given in Section 5.

Notations. In this paper, $R^{n}$ denotes the *n*-dimensional Euclidean space. $I_{n}$ denotes *n* dimensional unit matrix. For $q={\left[q_{1},q_{2},\ldots ,q_{N}\right]}^{T}$, ${∥q∥}_{1}$, ∥q∥ and ${∥q∥}_{\infty }$ represent the 1-norm, 2-norm and *∞*-norm of *q*, respectively. $\mathrm{sgn}\left(q\right)=[\mathrm{sgn}\left(q_{1}\right),\ldots ,$$\mathrm{sgn}\left(q_{N}\right){]}^{T}$, ${\mathrm{sig}}^{\alpha }\left(q\right)=\left[\right|q_{1}{|}^{\alpha }\mathrm{sgn}\left(q_{1}\right),\ldots ,$$|q_{N}{{|}^{\alpha }\mathrm{sgn}\left(q_{N}\right)]}^{T}$ where α>0 is a constant, sgn(·) represents the sign function. For a matrix $A\in R^{N\times N}$, let $A^{T}$ represent its transpose, $\lambda_{\max}\left(A\right)$ and $\lambda_{\min}\left(A\right)$ represent the maximum eigenvalue and minimum eigenvalue of *A*, respectively. The symbol ⊗ denotes the Kronecker product of matrices. diag(·) represents the diagonal matrix.

## 2. Preliminaries

### 2.1. Algebraic Graph Theory

A graph that consists of *N* nodes is represented by G=(V,E,A) where $V=\{v_{1},\ldots ,v_{N}\}$ is the set of nodes, E denotes the edges set, in which (i,j)∈E if there exists an edge between $v_{i}$ and $v_{j}$. The weighted adjacency matrix is denoted as $A=\left[a_{ij}\right]\in R^{N\times N}$, where $a_{ij}>0$ if (j,i)∈E, and $a_{ij}=0$, otherwise. The set of neighbors of agent *i* is denoted by $N_{i}=\left\{j\in V:\left(j,i\right)\in E\right\}$. The graph G is called directed and strongly connected if there exists a directed path between each pair of nodes. The graph G contains a directed spanning tree if there exists at least one root. The Laplacian matrix $L={\left[l_{ij}\right]}_{N\times N}$ is defined by $l_{ij}=-a_{ij}$ for i≠j, and $l_{ii}=\sum_{j\neq i}^{N}a_{ij}$.

### 2.2. Definitions and Lemmas

Consider the following differential equation

(1) $$\begin{array}{cc} \; & \dot{x}\left(t\right)=f\left(x\left(t\right)\right),\;\;\;\;\;\;x\left(0\right)=x_{0}, \end{array}$$

where $x\left(t\right)\in R^{n}$, and $f:R^{n}\mapsto R^{n}$ is a nonlinear function with f(0)=0. The following definitions and lemmas are given.

**Definition** **1** ([37])**.** *For any solution $x(t,t_{0},x_{0})$ of system (1), if there exists a positive number $T(x_{0})$ such that $x(t,t_{0},x_{0})=0$ for all $t\geq t_{0}+T\left(x_{0}\right)$, then the solution x=0 is said to be globally uniformly finite-time stable. $T(x_{0})$ is called the settling time. Moreover, x=0 is said to be globally fixed-time stable if $T(x_{0})$ is independent of the initial value $x_{0}$*.

**Lemma** **1** ([38])**.** *For system (1), if there is a regular, positive definite and radially unbounded function $W\left(x\right):R^{n}\to R$ such that any solution of (1) satisfies the inequality*

$$\begin{array}{cc} \; & \dot{W}\left(x\left(t\right)\right)\leq -{\left(\tau W^{p}\left(x\left(t\right)\right)+\phi W^{q}\left(x\left(t\right)\right)\right)}^{\varrho },\;\;x\left(t\right)\in R^{n}\backslash 0, \end{array}$$

*where τ,ϕ,p,ϱ>0, q≥0, pϱ>1, qϱ<1, then solution x=0 of system (1) is fixed-time stable, and the settling time $T(x_{0})$ is estimated by*

$$\begin{array}{c} T\left(x_{0}\right)\leq \frac{1}{\phi^{\varrho }}{\left(\frac{\phi }{\tau }\right)}^{\frac{1-q\varrho }{p-q}}\left(\frac{1}{1-q\varrho }+\frac{1}{p\varrho -1}\right). \end{array}$$

### 2.3. Problem Formulation

Consider a FONMAS consisting of *N* followers and a virtual leader indexed by i=1,2,…,N and 0, respectively. The dynamics is described by

(2) $$\begin{array}{ccc} {\dot{x}}_{i}(t)=f(x_{i}(t))+u_{i}(t)+w_{i}(t), & & i=1,2,\ldots ,N,\\ {\dot{x}}_{0}(t)=f(x_{0}(t))+u_{0}\left(t\right), \end{array}$$

where $x_{i}\left(t\right)\in R^{n}$, $u_{i}\left(t\right)\in R^{n}$ and $w_{i}\left(t\right)\in R^{n}$ are the state, the bounded control input and the external disturbance of the *i*th agent, respectively. $f(x_{i}\left(t\right))$ is a nonlinear function, which represents the inherent dynamics. In addition, we assume that the external disturbance is bounded, which satisfies $∥w_{i}{\left(t\right)∥}_{\infty }\leq D<\infty$, for D>0. $x_{0}\left(t\right)\in R^{n}$ and $u_{0}\left(t\right)\in R^{n}$ are the state and the bounded control input of the leader, respectively. $f(x_{0}\left(t\right))$ is a nonlinear function, which also represents the inherent dynamics.

The communication topology among followers is expressed as directed graph $\hat{G}$, and the corresponding Laplacian matrix is described by the weighted matrix $\hat{L}$. We use $b_{i}$ to represent the communication weight between the leader and the *i*th agent, in which $b_{i}>0$ if the *i*th agent can receive information from the leader, $b_{i}=0$ otherwise. In addition, we denote $B=\mathrm{diag}(b_{0},\ldots ,b_{N})$.

**Definition 2.**  *For the FONMAS (2), the fixed-time leader-following consensus is achieved for any initial conditions, if the following equations hold*

$$\begin{array}{ccc} \; & \lim_{t\to T}∥x_{i}\left(t\right)-x_{0}\left(t\right)∥=0,\; & ∥x_{i}\left(t\right)-x_{0}\left(t\right)∥\equiv 0,\;\;t\geq T,\;\;\;i=1,2,\ldots ,N, \end{array}$$

*where T>0 is called the settling time*.

**Assumption 1.**  *For the nonlinear function f(·), there exists a positive constant $l_{1}>0$ such that*

(3) $$\begin{array}{cc} \; & ∥f\left(z_{1}\left(t\right)\right)-f\left(z_{2}\left(t\right)\right)∥\leq l_{1}∥z_{1}\left(t\right)-z_{2}\left(t\right)∥, \end{array}$$

*where $z_{1}\left(t\right),z_{2}\left(t\right)\in R^{n}$*.

**Assumption 2.**  *The communication between the leader and all followers is represented by graph G which contains a directed spanning tree with the leader as the root. In addition, the communication topology $\hat{G}$ is directed.*

## 3. Main Results

### 3.1. Fixed-Time Consensus with Single Leader

In this section, in order to achieve consensus between leader and followers, the integral SMC protocol will be designed for FONMAS described by (2). Before moving on, we define the following error variables

(4) $$\begin{array}{cc} \; & {\tilde{x}}_{i}\left(t\right)=x_{i}\left(t\right)-x_{0}\left(t\right),\\ \; & {\tilde{u}}_{i}\left(t\right)=u_{i}\left(t\right)-u_{0}\left(t\right),\;\;\;\;\;\;\;\;\;\;i=1,2,\ldots ,N. \end{array}$$

Since the disturbances exist in the follower agent dynamics, the integral SMC technique is applied. Then, we define the following integral type sliding mode variable

(5) $$\begin{array}{cc} \; & \sigma_{i}\left(t\right)={\tilde{x}}_{i}\left(t\right)-\int_{0}^{t}\left(\chi_{i}^{\eta }\left(s\right)+\mathrm{sgn}\left(\chi_{i}\left(s\right)\right)\right)ds,\;\;\;\;\;\;\;\;\;\;i=1,2,\ldots ,N, \end{array}$$

where $\sigma_{i}\left(t\right)={\left[\sigma_{i1}\left(t\right),\sigma_{i2}\left(t\right),\ldots ,\sigma_{in}\left(t\right)\right]}^{T}$, $\chi_{i}\left(t\right)=-\left[\sum_{j\in N_{i}}a_{ij}\left({\tilde{x}}_{i}\left(t\right)-{\tilde{x}}_{j}\left(t\right)\right)+b_{i}\left({\tilde{x}}_{i}\left(t\right)\right)\right]$, and $\mathrm{sgn}\left(\chi_{i}\left(t\right)\right)={\left[\mathrm{sgn}\left(\chi_{i1}\left(t\right)\right),\mathrm{sgn}\left(\chi_{i2}\left(t\right)\right),\ldots ,\mathrm{sgn}\left(\chi_{in}\left(t\right)\right)\right]}^{T}$. η is the ratio of two positive odd numbers and η>1. When the sliding mode surface is reached, $\sigma_{i}\left(t\right)=0$ and ${\dot{\sigma }}_{i}\left(t\right)=0$. Hence, it has

(6) $$\begin{array}{cc} \; & {\dot{\tilde{x}}}_{i}\left(t\right)=\chi_{i}^{\eta }\left(t\right)+\mathrm{sgn}\left(\chi_{i}\left(t\right)\right),\;\;\;\;\;\;\;\;\;\;i=1,2,\ldots ,N. \end{array}$$

In order to reduce the control cost and increase the rate of convergence, the event-triggered consensus protocol is designed as follows

(7) $$\begin{array}{cc} {\tilde{u}}_{i}\left(t\right) & =\chi_{i}^{\eta }\left(t_{k}^{i}\right)+\mathrm{sgn}\left(\chi_{i}\left(t_{k}^{i}\right)\right)-K\mathrm{sgn}\left(\sigma_{i}\left(t_{k}^{i}\right)\right)-K_{3}{\mathrm{sig}}^{\beta +1}\left(\sigma_{i}\left(t_{k}^{i}\right)\right)\\ \; & -K_{4}∥{\tilde{x}}_{i}\left(t_{k}^{i}\right)∥\mathrm{sgn}\left(\sigma_{i}\left(t_{k}^{i}\right)\right),\;\;\;\;\;t\in \left[t_{k}^{i},t_{k+1}^{i}\right), \end{array}$$

where β>0, $K=K_{1}+K_{2}$, $K_{1},K_{2},K_{3},K_{4}$ are constants to be determined. $t_{k}^{i}$ is the triggering instant. Then, the novel measurement error is designed as

(8) $$\begin{array}{cc} e_{i}\left(t\right) & =\chi_{i}^{\eta }\left(t_{k}^{i}\right)+\mathrm{sgn}\left(\chi_{i}\left(t_{k}^{i}\right)\right)-K\mathrm{sgn}\left(\sigma_{i}\left(t_{k}^{i}\right)\right)-K_{3}{\mathrm{sig}}^{\beta +1}\left(\sigma_{i}\left(t_{k}^{i}\right)\right)\\ \; & -K_{4}∥{\tilde{x}}_{i}\left(t_{k}^{i}\right)∥\mathrm{sgn}\left(\sigma_{i}\left(t_{k}^{i}\right)\right)-(\chi_{i}^{\eta }\left(t\right)+\mathrm{sgn}\left(\chi_{i}\left(t\right)\right)-K\mathrm{sgn}\left(\sigma_{i}\left(t\right)\right)\\ \; & -K_{3}{\mathrm{sig}}^{\beta +1}\left(\sigma_{i}\left(t\right)\right)-K_{4}∥{\tilde{x}}_{i}\left(t\right)∥\mathrm{sgn}\left(\sigma_{i}\left(t\right)\right)). \end{array}$$

In this paper, a distributed event-triggered sampling control is proposed. The trigger instant of each agent only depends on its trigger function. Based on the zero order hold, the control input is a constant in each trigger interval. In order to make FONMAS (2) achieve leader-following consensus under the proposed protocol (7), the following theorem is given.

**Theorem 1.**  *Suppose that Assumptions 1 and 2 hold for the FONMAS (2). Under the protocol (7), the leader-following consensus can be achieved in fixed-time, if the following conditions are satisfied*

(9) $$\begin{array}{c} K_{1}\geq D,K_{2}>\max_{1\leq i\leq N}\left\{\xi_{i}\right\},K_{3}>0,K_{4}\geq l_{1}, \end{array}$$

*where $\xi_{i}>0$ for i=1,2,…,N. The triggering condition is defined as*

(10) $$\begin{array}{c} t_{k+1}^{i}=\inf\{t>t_{k}^{i}|∥e_{i}\left(t\right)∥-\xi_{i}\geq 0\},\;\;i=1,2,\ldots ,N. \end{array}$$

**Proof.**  Firstly, we prove that the sliding mode surface $\sigma_{i}\left(t\right)={\dot{\sigma }}_{i}\left(t\right)=0$ for i=1,2,…,N can be achieved in fixed-time. Consider the Lyapunov function as

(11) $$\begin{array}{cc} \; & V_{i}\left(t\right)=\frac{1}{2}\sigma_{i}^{T}\left(t\right)\sigma_{i}\left(t\right),\;\;\;\;\;\;i=1,2,\ldots ,N. \end{array}$$

Take the time derivative of $V_{i}\left(t\right)$ for $t\in [t_{k}^{i},t_{k+1}^{i})$, we have

(12) $$\begin{array}{cc} {\dot{V}}_{i}\left(t\right) & =\sigma_{i}^{T}\left(t\right){\dot{\sigma }}_{i}\left(t\right)\\ \; & =\sigma_{i}^{T}\left(t\right)\left({\dot{\tilde{x}}}_{i}\left(t\right)-\chi_{i}^{\eta }\left(t\right)-\mathrm{sgn}\left(\chi_{i}\left(t\right)\right)\right)\\ \; & =\sigma_{i}^{T}\left(t\right)\left({\dot{x}}_{i}\left(t\right)-{\dot{x}}_{0}\left(t\right)-\chi_{i}^{\eta }\left(t\right)-\mathrm{sgn}\left(\chi_{i}\left(t\right)\right)\right)\\ \; & =\sigma_{i}^{T}\left(t\right)\left(f\left(x_{i}\left(t\right)\right)+u_{i}\left(t\right)+w_{i}\left(t\right)-f\left(x_{0}\left(t\right)\right)-u_{0}\left(t\right)-\chi_{i}^{\eta }\left(t\right)-\mathrm{sgn}\left(\chi_{i}\left(t\right)\right)\right)\\ \; & =\sigma_{i}^{T}\left(t\right)\left(f\left(x_{i}\left(t\right)\right)-f\left(x_{0}\left(t\right)\right)+{\tilde{u}}_{i}\left(t\right)+w_{i}\left(t\right)-\chi_{i}^{\eta }\left(t\right)-\mathrm{sgn}\left(\chi_{i}\left(t\right)\right)\right)\\ \; & =\sigma_{i}^{T}\left(t\right)(f\left(x_{i}\left(t\right)\right)-f\left(x_{0}\left(t\right)\right)+e_{i}\left(t\right)+w_{i}\left(t\right)-K\mathrm{sgn}\left(\sigma_{i}\left(t\right)\right)\\ \; & -K_{3}{\mathrm{sig}}^{\beta +1}\left(\sigma_{i}\left(t\right)\right)-K_{4}∥{\tilde{x}}_{i}\left(t\right)∥\mathrm{sgn}\left(\sigma_{i}\left(t\right)\right)). \end{array}$$

Based on Assumption 1, it has

$$\begin{array}{cc} \; & \sigma_{i}^{T}\left(t\right)\left(f\left(x_{i}\left(t\right)\right)-f\left(x_{0}\left(t\right)\right)\right)\leq ∥\sigma_{i}\left(t\right)∥l_{1}∥x_{i}\left(t\right)-x_{0}\left(t\right)∥\leq l_{1}∥\sigma_{i}\left(t\right)∥∥{\tilde{x}}_{i}\left(t\right)∥,\\ \; & \sigma_{i}^{T}\left(t\right)\left(w_{i}\left(t\right)-K_{1}\mathrm{sgn}\left(\sigma_{i}\left(t\right)\right)\right)\leq D∥\sigma_{i}{\left(t\right)∥}_{1}-K_{1}{∥\sigma_{i}\left(t\right)∥}_{1}. \end{array}$$

Based on conditions (9), we can get

(13) $$\begin{array}{cc} \; & {\dot{V}}_{i}\left(t\right)\leq ∥e_{i}\left(t\right)∥∥\sigma_{i}\left(t\right)∥-K_{3}∥\sigma_{i}{\left(t\right)∥}^{\beta +2}-K_{2}∥\sigma_{i}\left(t\right)∥. \end{array}$$

According to triggering condition (10), we have

(14) $$\begin{array}{cc} {\dot{V}}_{i}\left(t\right) & \leq -\left(K_{2}-\xi_{i}\right)∥\sigma_{i}\left(t\right)∥-K_{3}{∥\sigma_{i}\left(t\right)∥}^{\beta +2}\\ \; & =-\left(K_{2}-\xi_{i}\right){\left(2V_{i}\left(t\right)\right)}^{\frac{1}{2}}-K_{3}{\left(2V_{i}\left(t\right)\right)}^{\frac{\beta +2}{2}}. \end{array}$$

The closed-loop system will get to the sliding mode surface in fixed-time, which can be obtained according to Lemma 1. The settling time can be computed as

(15) $$\begin{array}{cc} \; & T_{i}\leq \frac{1}{\sqrt{2}\left(K_{2}-\xi_{i}\right)}{\left(\frac{K_{2}-\xi_{i}}{K_{3}2^{\frac{\beta +1}{2}}}\right)}^{\frac{1}{\beta +1}}\left(2+\frac{2}{\beta }\right). \end{array}$$

Define $T=\max_{1\leq i\leq N}\left\{T_{i}\right\}$. Then, it is proved that the sliding mode surface $\sigma_{i}\left(t\right)=0$ can be reached for any t>T.

Secondly, we will prove that the leader-following consensus can be achieved in fixed-time. For convenience, $\chi_{i}\left(t\right)$ for i=1,2,…,N can be rewritten in the following compact form $\chi \left(t\right)=-\left(\left(\hat{L}+B\right)⊗I_{n}\right)\tilde{x}\left(t\right)$ and $∥\mathrm{sgn}\left(\chi \left(t\right)\right)∥\leq \sqrt{Nn}$.

Let $\hat{L}+B=H$. Based on Assumption 2, the matrix *H* is invertible and all eigenvalues have positive real parts. Therefore, there exists a positive symmetric matrix *P* such that $Q=PH+H^{T}P>0$. Define the Lyapunov function as $\tilde{V}\left(t\right)=\chi^{T}\left(t\right)\left(P⊗I_{n}\right)\chi \left(t\right)$, then taking the time derivative of $\tilde{V}\left(t\right)$ for t>T yields

(16) $$\begin{array}{cc} \dot{\tilde{V}}\left(t\right) & =-2\chi^{T}\left(t\right)\left(P⊗I_{n}\right)\left(H⊗I_{n}\right)\left(\chi^{\eta }\left(t\right)+\mathrm{sgn}\left(\chi \left(t\right)\right)\right)\\ \; & =-\chi^{T}\left(t\right)\left(Q⊗I_{n}\right)\chi^{\eta }\left(t\right)-\chi^{T}\left(t\right)\left(Q⊗I_{n}\right)\mathrm{sgn}\left(\chi \left(t\right)\right)\\ \; & \leq -\lambda_{\min}{\left(Q\right)∥\chi ∥}^{\eta +1}-\lambda_{\min}\left(Q\right)∥\chi ∥\\ \; & \leq -\frac{\lambda_{\min}\left(Q\right)}{\lambda_{\max}\left(P\right)}{\tilde{V}}^{\frac{\eta +1}{2}}\left(t\right)-\frac{\lambda_{\min}\left(Q\right)}{\lambda_{\max}\left(P\right)}{\tilde{V}}^{\frac{1}{2}}\left(t\right). \end{array}$$

By Lemma 1, we can conclude that the closed-loop system will achieve consensus in fixed-time. The settling time can be computed as

(17) $$\begin{array}{cc} \; & \tilde{T}\leq T+\frac{\lambda_{\max}\left(P\right)}{\lambda_{\min}\left(Q\right)}\left(2+\frac{2}{\eta -1}\right). \end{array}$$

□

**Remark** **1.** *In this paper, the general directed network topology is considered, so the matrix H is asymmetric. We need to select the positive definite matrix P to make it symmetric. In particular, if the network topology $\hat{G}$ is undirected, the matrix P corresponds to the identity matrix, and the construction of Lyapunov function $\tilde{V}\left(t\right)$ can be simplified. This reduces the computational burden.*

**Remark** **2.** *In [12,13,14], the finite-time consensus problem of MASs was studied. Compared with these literatures, we propose a fixed-time consensus protocol. Based on (17), we can find that the estimation of settling time is independent of initial values. In [15,16], the fixed-time consensus of MASs under ideal environment was studied. However, this paper considers a more complex environment in which agents of MASs are affected by external disturbances. We propose a new fixed-time consensus protocol based on integral sliding mode technique, which can suppress the disturbances better and improve the closed-loop performance of the system.*

**Remark** **3.** *There are generally three methods to deal with disturbances, namely internal made method, disturbances observation and sliding mode control. In [27,28], the disturbance rejection method was applied to eliminate the influence of disturbances in the protocols. However, in this paper, we adopt the integral sliding mode technique combined with event-triggered to suppress disturbances. Our research enriches the design method of control protocol and theoretical results. In [35,36], although the consensus of FONMASs with external disturbances was discussed by using integral sliding mode technique, only finite-time convergence was analyzed, and the estimation of settling time related to the initial conditions of system. To overcome this disadvantage, this paper proposes a new fixed-time event-triggered integral SMC protocol, in which the sliding mode surface can be reached and the consensus can be achieved in fixed-time.*

**Theorem 2.**  *Consider the FONMAS (2) with the event-triggered control protocol (7). If the triggering condition is defined by (10) and the conditions of Theorem 1 hold, then the Zeno behavior can be eliminated.*

**Proof.**  The proof is divided into two parts, before and after reaching the sliding mode surface.

On the one hand, we show the Zeno behavior does not exist before the systems achieve the sliding mode surface. Through the analysis of Theorem 1, the sliding mode surface will be reached when t>T. Therefore, we need to eliminate the Zeno behavior in the closed interval t∈[0,T]. Since $\chi_{i}\left(t\right)$ is a continuous function, it must exist a maximum value. Define $\tau_{i}=\max_{0\leq t\leq T}\{∥\chi_{i}^{\eta }\left(t\right)∥\}$ and $\phi_{i}=\max_{0\leq t\leq T}\{∥\mathrm{diag}\left(\chi_{i}^{\eta -1}\left(t\right)\right)∥\}$.

Take the time derivative of $∥e_{i}\left(t\right)∥$, it has

(18) $$\begin{array}{cc} \frac{d}{dt}∥e_{i}\left(t\right)∥\leq & ∥\frac{d}{dt}[\chi_{i}^{\eta }\left(t\right)+\mathrm{sgn}\left(\chi_{i}\left(t\right)\right)-K\mathrm{sgn}\left(\sigma_{i}\left(t\right)\right)-K_{3}{\mathrm{sig}}^{\beta +1}\left(\sigma_{i}\left(t\right)\right)\\ \; & -K_{4}∥{\tilde{x}}_{i}\left(t\right)∥\mathrm{sgn}\left(\sigma_{i}\left(t\right)\right)]∥\\ \leq & ∥\frac{d}{dt}\chi_{i}^{\eta }\left(t\right)∥+∥\frac{d}{dt}K_{3}{\mathrm{sig}}^{\beta +1}\left(\sigma_{i}\left(t\right)\right)∥+∥\frac{d}{dt}(K_{4}∥{\tilde{x}}_{i}\left(t\right)∥\mathrm{sgn}\left(\sigma_{i}\left(t\right)\right))∥\\ \leq & \eta ∥\mathrm{diag}\left(\chi_{i}^{\eta -1}\left(t\right)\right)∥∥{\dot{\chi }}_{i}\left(t\right)∥+K_{3}\left(\beta +1\right)∥\mathrm{diag}\left(\sigma_{i}^{\beta }\left(t\right)\right)∥∥{\dot{\sigma }}_{i}\left(t\right)∥+K_{4}\sqrt{n}∥{\dot{\tilde{x}}}_{i}\left(t\right)∥\\ \leq & \left[\eta \phi_{i}\sqrt{Nn}H_{ii}+K_{3}\left(\beta +1\right)∥\mathrm{diag}\left(\sigma_{i}^{\beta }\left(0\right)\right)∥+K_{4}\sqrt{n}\right]∥{\dot{\tilde{x}}}_{i}\left(t\right)∥+K_{3}\left(\beta +1\right)\tau_{i}\\ \; & \times ∥\mathrm{diag}\left(\sigma_{i}^{\beta }\left(0\right)\right)∥+K_{3}\left(\beta +1\right)\sqrt{n}∥\mathrm{diag}\left(\sigma_{i}^{\beta }\left(0\right)\right)∥\\ \leq & R_{i}l_{1}∥{\tilde{x}}_{i}\left(t\right)∥+R_{i}D+R_{i}∥{\tilde{u}}_{i}\left(t\right)∥+K_{3}\left(\beta +1\right)\tau_{i}∥\mathrm{diag}\left(\sigma_{i}^{\beta }\left(0\right)\right)∥\\ \; & +K_{3}\left(\beta +1\right)\sqrt{n}∥\mathrm{diag}\left(\sigma_{i}^{\beta }\left(0\right)\right)∥\\ \leq & R_{i}l_{1}{\bar{x}}_{i}+R_{i}D+R_{i}{\bar{u}}_{i}+K_{3}\left(\beta +1\right)\tau_{i}∥\mathrm{diag}\left(\sigma_{i}^{\beta }\left(0\right)\right)∥\\ \; & +K_{3}\left(\beta +1\right)\sqrt{n}∥\mathrm{diag}\left(\sigma_{i}^{\beta }\left(0\right)\right)∥, \end{array}$$

where $R_{i}=\eta \phi_{i}\sqrt{Nn}H_{ii}+K_{3}\left(\beta +1\right)∥\mathrm{diag}\left(\sigma_{i}^{\beta }\left(0\right)\right)∥+K_{4}\sqrt{n}$, $H_{ii}$ is the element of *i*-th row and column of matrix *H*, ${\bar{x}}_{i}=\max_{0\leq t\leq T}\{∥{\tilde{x}}_{i}\left(t\right)∥\}$ and ${\bar{u}}_{i}=\max_{0\leq t\leq T}\{∥{\tilde{u}}_{i}\left(t\right)∥\}$. Combination with $e_{i}\left(t_{k}^{i}\right)=0$, it yields

(19) $$\begin{array}{cc} ∥e_{i}\left(t\right)∥\leq & [R_{i}l_{1}{\bar{x}}_{i}+R_{i}D+R_{i}{\bar{u}}_{i}+K_{3}\left(\beta +1\right)\tau_{i}∥\mathrm{diag}\left(\sigma_{i}^{\beta }\left(0\right)\right)∥\\ \; & +K_{3}\left(\beta +1\right)\sqrt{n}∥\mathrm{diag}\left(\sigma_{i}^{\beta }\left(0\right)\right)∥]\left(t-t_{k}^{i}\right). \end{array}$$

Using the triggering condition (10), the next trigger instant satisfies $∥e_{i}\left(t_{k+1}^{i}\right)∥=\xi_{i}$. Therefore,

(20) $$\begin{array}{cc} \xi_{i}\leq & [R_{i}l_{1}{\bar{x}}_{i}+R_{i}D+R_{i}{\bar{u}}_{i}+K_{3}\left(\beta +1\right)\tau_{i}∥\mathrm{diag}\left(\sigma_{i}^{\beta }\left(0\right)\right)∥\\ \; & +K_{3}\left(\beta +1\right)\sqrt{n}∥\mathrm{diag}\left(\sigma_{i}^{\beta }\left(0\right)\right)∥]\left(t_{k+1}^{i}-t_{k}^{i}\right). \end{array}$$

Denote $Q_{1i}=R_{i}l_{1}{\bar{x}}_{i}+R_{i}D+R_{i}{\bar{u}}_{i}+K_{3}\left(\beta +1\right)\tau_{i}∥\mathrm{diag}\left(\sigma_{i}^{\beta }\left(0\right)\right)∥+K_{3}\left(\beta +1\right)\sqrt{n}∥\mathrm{diag}\left(\sigma_{i}^{\beta }\left(0\right)\right)∥$, and $\Delta T_{k}^{i}=t_{k+1}^{i}-t_{k}^{i}$, we can get $\Delta T_{k}^{i}\geq \frac{\xi_{i}}{Q_{1i}}>0$.

On the other hand, when the sliding mode surface is reached, $\sigma_{i}\left(t\right)=0$. Similar to the above proof, we can obtain

(21) $$\begin{array}{cc} \frac{d}{dt}∥e_{i}\left(t\right)∥\leq & ∥\frac{d}{dt}[\chi_{i}^{\eta }\left(t\right)+\mathrm{sgn}\left(\chi_{i}\left(t\right)\right)-K\mathrm{sgn}\left(\sigma_{i}\left(t\right)\right)-K_{3}{\mathrm{sig}}^{\beta +1}\left(\sigma_{i}\left(t\right)\right)\\ & -K_{4}∥{\tilde{x}}_{i}\left(t\right)∥\mathrm{sgn}\left(\sigma_{i}\left(t\right)\right)]∥\\ \leq & \eta ∥\mathrm{diag}\left(\chi_{i}^{\eta -1}\left(t\right)\right)∥∥{\dot{\chi }}_{i}\left(t\right)∥\\ \leq & \eta \phi_{i}\sqrt{Nn}H_{ii}{\left(\frac{1}{\lambda_{\min}\left(P\right)}\right)}^{\frac{\eta }{2}}\tilde{V}{\left(0\right)}^{\frac{\eta }{2}}+\eta \phi_{i}NnH_{ii}. \end{array}$$

Combination with $e_{i}\left(t_{k}^{i}\right)=0$, one has

(22) $$\begin{array}{cc} ∥e_{i}\left(t\right)∥\leq & \left[\eta \phi_{i}\sqrt{Nn}H_{ii}{\left(\frac{1}{\lambda_{\min}\left(P\right)}\right)}^{\frac{\eta }{2}}\tilde{V}{\left(0\right)}^{\frac{\eta }{2}}+\eta \phi_{i}NnH_{ii}\right]\left(t-t_{k}^{i}\right). \end{array}$$

Using the triggering condition (10), on can obtain

(23) $$\begin{array}{c} \Delta T_{k}^{i}\geq \frac{\xi_{i}}{Q_{2i}}>0, \end{array}$$

where $Q_{2i}=\eta \phi_{i}\sqrt{Nn}H_{ii}{\left(\frac{1}{\lambda_{\min}\left(P\right)}\right)}^{\frac{\eta }{2}}\tilde{V}{\left(0\right)}^{\frac{\eta }{2}}+\eta \phi_{i}NnH_{ii}$, and $\Delta T_{k}^{i}=t_{k+1}^{i}-t_{k}^{i}$. Based on the above analysis, the Zeno behavior can be avoided in control process. □

**Remark 4.**  *Since the trigger mechanism exists in the whole control process, the proof of Theorem 2 divided into two parts, i.e., before and after the system reaches the sliding mode surface. In this paper, a static distributed event-triggered strategy is developed. In order to reduce the number of triggers more effectively, we will consider the dynamic event-triggered control strategy in our future work.*

### 3.2. Fixed-Time Containment Control with Multiple Leaders

In this section, we consider the MASs with multiple leaders. The main aim is to make MASs realize containment control in fixed-time by designing appropriate control protocol. That means all follower agents’ states converge to the convex combination of leaders’ states in fixed-time. In particular, if the MASs has only one leader, the containment control will degenerate into leader-following consensus.

For the sake of generality, we hypothesize that the FONMAS consisting of *N* followers and *M* leaders indexed by indexed by i=1,…,N and j=N+1,…,N+M, respectively. The dynamics of the FONMAS is described by

(24) $$\begin{array}{cc} \; & {\dot{x}}_{i}\left(t\right)=f\left(x_{i}\left(t\right)\right)+u_{i}\left(t\right)+w_{i}\left(t\right),\;\;\;\;\;\;\;\;\;\;i=1,\ldots ,N,\\ \; & {\dot{x}}_{j}\left(t\right)=f\left(x_{j}\left(t\right)\right)+u_{j}\left(t\right)+w_{j}\left(t\right),\;\;\;\;\;\;\;\;\;j=N+1,\ldots ,N+M, \end{array}$$

where $x_{i}\left(t\right)\in R^{n}$, $u_{i}\left(t\right)\in R^{n}$ and $w_{i}\left(t\right)\in R^{n}$ are the state, the bounded control input and the external disturbance of the *i*th agent, respectively. $f(x_{i}\left(t\right))$ is a nonlinear function which represents the inherent dynamics. $x_{j}\left(t\right)\in R^{n}$, $u_{j}\left(t\right)\in R^{n}$ and $w_{j}\left(t\right)\in R^{n}$ are the state, the bounded control input and the internal disturbance of the *j*th leader, respectively. $f(x_{j}\left(t\right))$ is a nonlinear function, which also represents the inherent dynamics. In addition, we assume that the disturbances are bounded, which satisfy $∥w_{i}{\left(t\right)∥}_{\infty }\leq B<\infty$, $∥w_{j}{\left(t\right)∥}_{\infty }\leq F<\infty$ for B>0 and F>0.

**Assumption 3.**  *Suppose that the communication among the leaders and followers is represented by graph G. For each follower, there exists at least one leader that has a directed path to it.*

**Assumption 4.**  *Given scalars $\rho_{1},\rho_{2},\ldots ,\rho_{M}$, satisfying $\sum_{j=1}^{M}\rho_{j}=1$ and $\rho_{j}\geq 0$. There exists a constant $l_{2}>0$ such that for $x_{i}\left(t\right),x_{j}\left(t\right)\in R^{n}$*,

$$\begin{array}{c} ∥f\left(x_{i}\left(t\right)\right)-\sum_{j=1}^{M}\rho_{j}f\left(x_{j}\left(t\right)\right)∥\leq l_{2}∥x_{i}\left(t\right)-\sum_{j=1}^{M}\rho_{j}x_{j}\left(t\right)∥. \end{array}$$

Under Assumption 3, the Laplcain matrix of graph *G* is denoted by *L*, which can be decomposed into $L=\left[\begin{array}{cc} L_{1} & L_{2}\\ 0 & 0 \end{array}\right]$, where $L_{1}$ is a nonsingular matrix, $L_{2}\in R^{N\times M}$ has at least one positive entry and $-L_{1}^{-1}L_{2}1_{M\times 1}=1_{N\times 1}$.

Before moving on, we define the following error variables

(25) $$\begin{array}{c} \tilde{X}\left(t\right)=\left(L_{1}⊗I_{n}\right)X_{1}\left(t\right)+\left(L_{2}⊗I_{n}\right)X_{2}\left(t\right),\\ \tilde{U}\left(t\right)=\left(L_{1}⊗I_{n}\right)U_{1}\left(t\right)+\left(L_{2}⊗I_{n}\right)U_{2}\left(t\right),\\ \tilde{W}\left(t\right)=\left(L_{1}⊗I_{n}\right)W_{1}\left(t\right)+\left(L_{2}⊗I_{n}\right)W_{2}\left(t\right), \end{array}$$

where

$$\begin{array}{c} \tilde{X}\left(t\right)={\left[{\tilde{X}}_{1}^{T}\left(t\right),{\tilde{X}}_{2}^{T}\left(t\right),\ldots ,{\tilde{X}}_{N}^{T}\left(t\right)\right]}^{T},\;\tilde{U}\left(t\right)={\left[{\tilde{U}}_{1}^{T}\left(t\right),{\tilde{U}}_{2}^{T}\left(t\right),\ldots ,{\tilde{U}}_{N}^{T}\left(t\right)\right]}^{T},\;\tilde{W}\left(t\right)={\left[{\tilde{W}}_{1}^{T}\left(t\right),\ldots ,{\tilde{W}}_{N}^{T}\left(t\right)\right]}^{T},\\ X_{1}\left(t\right)={\left[x_{1}^{T}\left(t\right),\ldots ,x_{N}^{T}\left(t\right)\right]}^{T},X_{2}\left(t\right)={\left[x_{N+1}^{T}\left(t\right),\ldots ,x_{N+M}^{T}\left(t\right)\right]}^{T},\\ U_{1}\left(t\right)={\left[u_{1}^{T}\left(t\right),u_{2}^{T}\left(t\right),\ldots ,u_{N}^{T}\left(t\right)\right]}^{T},U_{2}\left(t\right)={\left[u_{N+1}^{T}\left(t\right),u_{N+2}^{T}\left(t\right),\ldots ,u_{N+M}^{T}\left(t\right)\right]}^{T},\\ W_{1}\left(t\right)={\left[w_{1}^{T}\left(t\right),w_{2}^{T}\left(t\right),\ldots ,w_{N}^{T}\left(t\right)\right]}^{T},W_{2}\left(t\right)={\left[w_{N+1}^{T}\left(t\right),w_{N+2}^{T}\left(t\right),\ldots ,w_{N+M}^{T}\left(t\right)\right]}^{T}. \end{array}$$

Combination with Assumption 3 and the property of Laplacian matrix *L*, we can easily obtain that the containment control is achieve in fixed-time if and only if there exists a T>0 such that $\lim_{t\to T}∥\tilde{X}\left(t\right)∥=0$ and $∥\tilde{X}\left(t\right)∥\equiv 0$ for t>T.

Considering the disturbances in the system, the consensus protocol can employ sliding mode approach. The integral type sliding variable is defined as follows

(26) $$\begin{array}{c} \sigma_{i}\left(t\right)={\tilde{X}}_{i}\left(t\right)-\int_{0}^{t}\left({\tilde{\chi }}_{i}^{\eta }\left(s\right)+\mathrm{sgn}\left({\tilde{\chi }}_{i}\left(s\right)\right)\right)ds, \end{array}$$

where ${\tilde{\chi }}_{i}\left(t\right)=-{\tilde{X}}_{i}\left(t\right)$, η is the ratio of two positive odd numbers and η>1. The sliding mode manifold (26) is given by following comport form

(27) $$\begin{array}{cc} \; & \sigma \left(t\right)=\tilde{X}\left(t\right)-\int_{0}^{t}\left({\tilde{\chi }}^{\eta }\left(s\right)+\mathrm{sgn}\left(\tilde{\chi }\left(s\right)\right)\right)ds. \end{array}$$

When the sliding mode surface is reached, σ(t)=0 and $\dot{\sigma }\left(t\right)=0$. Hence, it has

(28) $$\begin{array}{cc} \; & \dot{\tilde{X}}\left(t\right)={\tilde{\chi }}^{\eta }\left(t\right)+\mathrm{sgn}\left(\tilde{\chi }\left(t\right)\right). \end{array}$$

In order to reduce the control cost and increase the rate of convergence, the event-triggered sample-data control protocol is presented as

(29) $$\begin{array}{cc} {\tilde{U}}_{i}\left(t\right)= & {\tilde{\chi }}_{i}^{\eta }\left(t_{k}\right)+\mathrm{sgn}\left({\tilde{\chi }}_{i}\left(t_{k}\right)\right)-K\mathrm{sgn}\left(\sigma_{i}\left(t_{k}\right)\right)-K_{3}{\mathrm{sig}}^{\beta +1}\left(\sigma_{i}\left(t_{k}\right)\right)\\ & -K_{4}∥\tilde{X}\left(t_{k}\right)∥\mathrm{sgn}\left(\sigma_{i}\left(t_{k}\right)\right),\;\;\;\;\;\;\;\;\;t\in \left[t_{k},t_{k+1}\right), \end{array}$$

where β>0, $K=K_{1}+K_{2}$, $K_{1},K_{2},K_{3},K_{4}$ are constants to be determined. $t_{k}$ is the triggering instant.

Similarly, the controller (29) can be rewritten in the following comport form

(30) $$\begin{array}{cc} \tilde{U}\left(t\right)= & {\tilde{\chi }}^{\eta }\left(t_{k}\right)+\mathrm{sgn}\left(\tilde{\chi }\left(t_{k}\right)\right)-K\mathrm{sgn}\left(\sigma \left(t_{k}\right)\right)-K_{3}{\mathrm{sig}}^{\beta +1}\left(\sigma \left(t_{k}\right)\right)\\ \; & -K_{4}∥\tilde{X}\left(t_{k}\right)∥\mathrm{sgn}\left(\sigma \left(t_{k}\right)\right),\;\;\;\;\;\;\;\;\;t\in \left[t_{k},t_{k+1}\right). \end{array}$$

Then, the novel measurement error for the system (24) is designed as

(31) $$\begin{array}{cc} e(t) & ={\tilde{\chi }}^{\eta }\left(t_{k}\right)+\mathrm{sgn}\left(\tilde{\chi }\left(t_{k}\right)\right)-K\mathrm{sgn}\left(\sigma \left(t_{k}\right)\right)-K_{3}{\mathrm{sig}}^{\beta +1}\left(\sigma \left(t_{k}\right)\right)\\ \; & -K_{4}∥\tilde{X}\left(t_{k}\right)∥\mathrm{sgn}\left(\sigma \left(t_{k}\right)\right)-({\tilde{\chi }}^{\eta }\left(t\right)+\mathrm{sgn}\left(\tilde{\chi }\left(t\right)\right)-K\mathrm{sgn}\left(\sigma \left(t\right)\right)\\ \; & -K_{3}{\mathrm{sig}}^{\beta +1}\left(\sigma \left(t\right)\right)-K_{4}∥\tilde{X}\left(t\right)∥\mathrm{sgn}\left(\sigma \left(t\right)\right)). \end{array}$$

**Theorem 3.**  *Suppose that Assumptions 3 and 4 hold for the FONMAS (24). Under the protocol (30), the containment control can be achieved in fixed-time, if the following inequalities are satisfied:*

(32) $$\begin{array}{cc} \; & K_{1}\geq ∥L_{1}∥B+∥L_{2}∥F,K_{2}>\xi ,K_{3}>0,K_{4}\geq l_{2}∥L_{1}∥∥L_{1}^{-1}∥. \end{array}$$

*The triggering condition is defined as*

(33) $$\begin{array}{cc} \; & t_{k+1}=\inf\{t>t_{k}|∥e\left(t\right)∥-\xi \geq 0\}, \end{array}$$

*where ξ>0*.

**Proof.**  Consider the Lyapunov function as

(34) $$\begin{array}{c} V\left(t\right)=\frac{1}{2}\sigma^{T}\left(t\right)\sigma \left(t\right). \end{array}$$

For $t\in [t_{k},t_{k+1})$, the derivative of V(t) is

(35) $$\begin{array}{cc} \dot{V}\left(t\right)= & \sigma^{T}\left(t\right)\dot{\sigma }\left(t\right)\\ = & \sigma^{T}\left(t\right)\left(\dot{\tilde{X}}\left(t\right)-{\tilde{\chi }}^{\eta }\left(t\right)-\mathrm{sgn}\left(\tilde{\chi }\left(t\right)\right)\right)\\ = & \sigma^{T}\left(t\right)\left(\left(L_{1}⊗I_{n}\right)F_{1}+\left(L_{2}⊗I_{n}\right)F_{2}+\tilde{U}\left(t\right)+\tilde{W}\left(t\right)-{\tilde{\chi }}^{\eta }\left(t\right)-\mathrm{sgn}\left(\tilde{\chi }\left(t\right)\right)\right)\\ = & \sigma^{T}\left(t\right)(\left(L_{1}⊗I_{n}\right)F_{1}+\left(L_{2}⊗I_{n}\right)F_{2}+e\left(t\right)+\tilde{W}\left(t\right)-K\mathrm{sgn}\left(\sigma \left(t\right)\right)\\ & -K_{3}{\mathrm{sig}}^{\beta +1}\left(\sigma \left(t\right)\right)-K_{4}∥\tilde{X}\left(t\right)∥\mathrm{sgn}\left(\sigma \left(t\right)\right)). \end{array}$$

Define $F_{1}={\left[f^{T}\left(x_{1}\left(t\right)\right),\ldots ,f^{T}\left(x_{N}\left(t\right)\right)\right]}^{T}$, $F_{2}={\left[f^{T}\left(x_{N+1}\left(t\right)\right),\ldots ,f^{T}\left(x_{N+M}\left(t\right)\right)\right]}^{T}$. Let $-L_{1}^{-1}L_{2}≜{\left(\rho_{1}^{T},\rho_{2}^{T},\ldots ,\rho_{N}^{T}\right)}^{T}$, where $\rho_{i}=\left(\rho_{i1},\ldots ,\rho_{iM}\right)$. From Assumption 4,

$$\begin{array}{cc} \; & ∥F_{1}+\left(L_{1}^{-1}L_{2}⊗I_{n}\right)F_{2}∥\\ \; & =∥{\left[{\left(f\left(x_{1}\left(t\right)\right)-\sum_{j=1}^{M}\rho_{1j}f\left(x_{j}\left(t\right)\right)\right)}^{T},\ldots ,{\left(f\left(x_{N}\left(t\right)\right)-\sum_{j=1}^{M}\rho_{Nj}f\left(x_{j}\left(t\right)\right)\right)}^{T}\right]}^{T}∥\\ \; & =∥\left(∥(f\left(x_{1}\left(t\right)\right)-\sum_{j=1}^{M}\rho_{1j}f\left(x_{j}\left(t\right)\right))∥,\ldots ,∥\left(f\left(x_{N}\left(t\right)\right)-\sum_{j=1}^{M}\rho_{Nj}f\left(x_{j}\left(t\right)\right)\right)∥\right)∥\\ \; & \leq ∥(l_{2}∥x_{1}\left(t\right)-\sum_{j=1}^{M}\rho_{1j}x_{j}\left(t\right)∥,\ldots ,l_{2}∥x_{N}\left(t\right)-\sum_{j=1}^{M}\rho_{Nj}x_{j}\left(t\right))∥∥\\ \; & =l_{2}∥\left(L_{1}^{-1}⊗I_{n}\right)\tilde{X}\left(t\right)∥\leq l_{2}∥L_{1}^{-1}∥∥\tilde{X}\left(t\right)∥, \end{array}$$

$$\begin{array}{cc} \; & \sigma^{T}\left(t\right)\left(\tilde{W}\left(t\right)-K_{1}\mathrm{sgn}\left(\sigma \left(t\right)\right)\right)\leq (∥L_{1}∥B+∥L_{2}{∥F)∥\sigma \left(t\right)∥}_{1}-K_{1}{∥\sigma \left(t\right)∥}_{1}. \end{array}$$

Based on (32), we can get

(36) $$\begin{array}{cc} \; & \dot{V}\left(t\right)\leq ∥e\left(t\right)∥∥\sigma \left(t\right)∥-K_{3}{∥\sigma \left(t\right)∥}^{\beta +2}-K_{2}∥\sigma \left(t\right)∥. \end{array}$$

According to (33), we have

(37) $$\begin{array}{cc} \dot{V}\left(t\right) & \leq -\left(K_{2}-\xi \right)∥\sigma \left(t\right)∥-K_{3}{∥\sigma \left(t\right)∥}^{\beta +2}\\ \; & =-\left(K_{2}-\xi \right){\left(2V\left(t\right)\right)}^{\frac{1}{2}}-K_{3}{\left(2V\left(t\right)\right)}^{\frac{\beta +2}{2}}. \end{array}$$

According to Lemma 1, the closed-loop system (24) will get to the sliding mode surface in fixed-time. The settling time can be estimated by

(38) $$\begin{array}{cc} \; & \bar{T}\leq \frac{1}{\sqrt{2}\left(K_{2}-\xi \right)}{\left(\frac{K_{2}-\xi }{K_{3}2^{\frac{\beta +1}{2}}}\right)}^{\frac{1}{\beta +1}}\left(2+\frac{2}{\beta }\right). \end{array}$$

Then, it is proved that σ(t)=0 is reached for $t>\bar{T}$.

Then, we will prove that the containment control can be achieved in fixed-time. Define the Lyapunov function as $\hat{V}\left(t\right)={\tilde{\chi }}^{T}\left(t\right)\tilde{\chi }\left(t\right)$. Taking the time derivative of $\hat{V}\left(t\right)$ for $t>\bar{T}$ yields

(39) $$\begin{array}{cc} \dot{\hat{V}}\left(t\right) & =-{\tilde{\chi }}^{T}\left(t\right)\left({\tilde{\chi }}^{\eta }\left(t\right)+\mathrm{sgn}\left(\tilde{\chi }\left(t\right)\right)\right)\\ \; & =-∥\tilde{\chi }{\left(t\right)∥}^{\eta +1}-{∥\tilde{\chi }\left(t\right)∥}_{1}\\ \; & \leq -{\hat{V}}^{\frac{\eta +1}{2}}\left(t\right)-{\hat{V}}^{\frac{1}{2}}\left(t\right). \end{array}$$

By Lemma 1, we can conclude that the closed-loop system will achieve containment control in fixed-time. The settling time can be computed as

(40) $$\begin{array}{cc} \hat{T}\leq & \bar{T}+(2+\frac{2}{\eta -1}). \end{array}$$

The proof is finished. □

**Remark** **5.** *In [27], the fixed-time consensus problem of MASs with nonlinear dynamics and indeterminate disturbances was considered based on event-triggered method. Compared with [27], we introduce the integral sliding mode technique to deal with disturbances, and consider the containment control problem in the case of multiple leaders. In addition, the event-triggered strategy applied in this paper can greatly save computation and communication resources.*

**Theorem 4.**  *Consider the FONMAS (24) with the event-triggered control protocol (30). If the triggering condition is defined by (33) and all conditions of Theorem 3 are satisfied, then the Zeno behavior can be avoided.*

**Proof.**  Similar to the proof of Theorem 2, the proof is divided into two parts.

First, we show that the Zeno behavior does not exist before the systems reach the sliding mode surface. Through the analysis of Theorem 3, we know that sliding mode surface will be reached when $t>\bar{T}$. Therefore, we need to eliminate the Zeno behavior in the closed interval $\left[0,\bar{T}\right]$. Since χ(t) is a continuous function, it must exist a maximum value. Define $\varepsilon =\max_{0\leq t\leq \bar{T}}\{∥{\tilde{\chi }}^{\eta }\left(t\right)∥\}$ and $\gamma =\max_{0\leq t\leq \bar{T}}\{∥\mathrm{diag}\left({\tilde{\chi }}^{\eta -1}\left(t\right)\right)∥\}$.

Take the time derivative of ∥e(t)∥, we have

(41) $$\begin{array}{cc} \frac{d}{dt}∥e\left(t\right)∥\leq & ∥\frac{d}{dt}[{\tilde{\chi }}^{\eta }\left(t\right)+\mathrm{sgn}\left(\tilde{\chi }\left(t\right)\right)-K\mathrm{sgn}\left(\sigma \left(t\right)\right)-K_{3}{\mathrm{sig}}^{\beta +1}\left(\sigma \left(t\right)\right)\\ \; & -K_{4}∥\tilde{X}\left(t\right)∥\mathrm{sgn}\left(\sigma \left(t\right)\right)]∥\\ \leq & \psi l_{2}∥L_{1}∥∥L_{1}^{-1}∥\bar{X}+\psi (∥L_{1}∥B+∥L_{2}∥F)+\psi \bar{U}+K_{3}\left(\beta +1\right)\varepsilon \\ \; & \times ∥\mathrm{diag}\left(\sigma^{\beta }\left(0\right)\right)∥+K_{3}\left(\beta +1\right)\sqrt{Nn}∥\mathrm{diag}\left(\sigma^{\beta }\left(0\right)\right)∥, \end{array}$$

where $\psi =\eta \gamma +K_{3}\left(\beta +1\right)∥\mathrm{diag}\left(\sigma^{\beta }\left(0\right)\right)∥+K_{4}\sqrt{Nn}$, $\bar{X}=\max_{0\leq t\leq \bar{T}}\{∥\tilde{X}\left(t\right)∥\}$ and $\bar{U}=\max_{0\leq t\leq \bar{T}}\{∥\tilde{U}\left(t\right)∥\}$. Based on $e(t_{k})=0$, it has

(42) $$\begin{array}{cc} ∥e(t)∥\leq & [\psi l_{2}∥L_{1}∥∥L_{1}^{-1}∥\bar{X}+\psi (∥L_{1}∥B+∥L_{2}∥F)+\psi \bar{U}+K_{3}\left(\beta +1\right)\varepsilon ∥\mathrm{diag}\left(\sigma^{\beta }\left(0\right)\right)∥\\ \; & +K_{3}\left(\beta +1\right)\sqrt{Nn}∥\mathrm{diag}\left(\sigma^{\beta }\left(0\right)\right)∥]\left(t-t_{k}\right). \end{array}$$

Applying the triggering mechanism (33), it has $∥e(t_{k+1})∥=\xi$. Therefore,

(43) $$\begin{array}{cc} \xi \leq & [\psi l_{2}∥L_{1}∥∥L_{1}^{-1}∥\bar{X}+\psi (∥L_{1}∥B+∥L_{2}∥F)+\psi \bar{U}+K_{3}\left(\beta +1\right)\varepsilon \\ \; & \times ∥\mathrm{diag}\left(\sigma^{\beta }\left(0\right)\right)∥+K_{3}\left(\beta +1\right)\sqrt{Nn}∥\mathrm{diag}\left(\sigma^{\beta }\left(0\right)\right)∥]\left(t_{k+1}-t_{k}\right). \end{array}$$

Denote $\pi_{1}=\psi l_{2}∥L_{1}∥∥L_{1}^{-1}∥\bar{X}+\psi (∥L_{1}∥B+∥L_{2}∥F)+\psi \bar{U}+K_{3}\left(\beta +1\right)\varepsilon ∥\mathrm{diag}\left(\sigma^{\beta }\left(0\right)\right)∥+K_{3}\left(\beta +1\right)\sqrt{Nn}∥\mathrm{diag}\left(\sigma^{\beta }\left(0\right)\right)∥$, and $\Delta T_{k}=\left(t_{k+1}-t_{k}\right)$, we can get $\Delta T_{k}\geq \frac{\xi }{\pi_{1}}>0$.

Next, we prove that the Zeno behavior can be avoided when the sliding mode surface is reached. Similar to the above proof, we can obtain

(44) $$\begin{array}{cc} \frac{d}{dt}∥e\left(t\right)∥\leq & ∥\frac{d}{dt}[{\tilde{\chi }}^{\eta }\left(t\right)+\mathrm{sgn}\left(\tilde{\chi }\left(t\right)\right)-K\mathrm{sgn}\left(\sigma \left(t\right)\right)-K_{3}{\mathrm{sig}}^{\beta +1}\left(\sigma \left(t\right)\right)\\ \; & -K_{4}∥\tilde{X}\left(t\right)∥\mathrm{sgn}\left(\sigma \left(t\right)\right)]∥\\ \leq & \eta \gamma \hat{V}{\left(0\right)}^{\frac{\eta }{2}}+\eta \gamma \sqrt{Nn}. \end{array}$$

Combination with $e(t_{k})=0$, it yields

(45) $$\begin{array}{cc} ∥e(t)∥\leq & \left[\eta \gamma \hat{V}{\left(0\right)}^{\frac{\eta }{2}}+\eta \gamma \sqrt{Nn}\right]\left(t-t_{k}\right). \end{array}$$

When the event next event is triggered, it has ∥e(t)∥=ξ. Therefore,

(46) $$\begin{array}{c} \xi \leq \left[\eta \gamma \hat{V}{\left(0\right)}^{\frac{\eta }{2}}+\eta \gamma \sqrt{Nn}\right]\left(t_{k+1}-t_{k}\right). \end{array}$$

Let $\pi_{2}=\eta \gamma \hat{V}{\left(0\right)}^{\frac{\eta }{2}}+\eta \gamma \sqrt{Nn}$ and $\Delta T_{k}=t_{k+1}-t_{k}$, we can get $\Delta T_{k}\geq \frac{\xi }{\pi_{2}}>0$. Based on above analysis, the lower bound of event-triggered interval is positive, then Zeno phenomenon is eliminated in the whole control process. □

## 4. Numerical Example

In this section, two numerical examples are presented to demonstrate the effectiveness of the control protocols.

**Figure 1:**
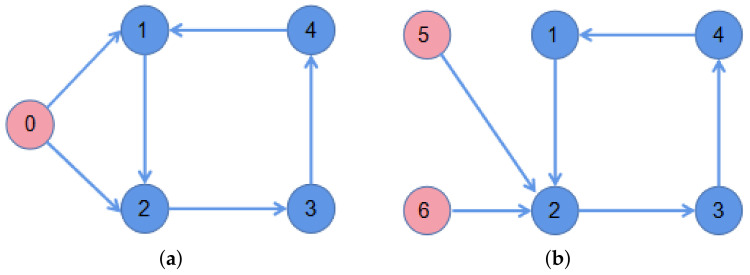
The network topology. (**a**) Topology with 1 leader. (**b**) Topology with 2 leaders.

**Example 1.**  *Consider the FONMAS (2) with one leader and four followers. Figure 1a shows the directed communication topology between the leader and all followers. Obviously, Assumption 2 is satisfied. The nonlinear function is defined as follows*

$$\begin{array}{c} f\left(x_{i}\left(t\right)\right)=\left(\begin{array}{c} -x_{i1}\left(t\right)+2g\left(x_{i1}\left(t\right)\right)-1.2g\left(x_{i2}\left(t\right)\right)\\ -x_{i2}\left(t\right)+1.2g\left(x_{i1}\left(t\right)\right)+2g\left(x_{i2}\left(t\right)\right) \end{array}\right),\;\;i=0,1,\ldots ,4. \end{array}$$

*where $g\left(x_{ij}\left(t\right)\right)=0.5(|x_{ij}\left(t\right)+1|-|x_{ij}\left(t\right)-1\left|\right)+0.01sgn\left(x_{ij}\left(t\right)\right),i=0,1,\ldots ,4,j=1,2.$ Then, Assumption 1 holds. The external disturbances are defined as $w_{11}\left(t\right)=w_{12}\left(t\right)=0.05\sin\left(t\right)+0.1\cos\left(t\right)$, $w_{21}\left(t\right)=w_{22}\left(t\right)=0.05\sin\left(t\right)+0.1\cos\left(t\right)$, $w_{31}\left(t\right)=w_{32}\left(t\right)=0.05\sin\left(t\right)$, and $w_{41}\left(t\right)=w_{42}\left(t\right)=0.1\cos\left(t\right)$. It follows that $∥w_{i}{\left(t\right)∥}_{\infty }\leq 0.2,i=1,2,3,4$. The control input of leader is $u_{01}\left(t\right)=u_{02}\left(t\right)=0.1\sin\left(t\right)+0.1\cos\left(t\right)$. We choose the controller parameters $K_{1}=0.2$, $K_{2}=1.7$, $K_{3}=1.5$, $K_{4}=2$, $\eta =\frac{7}{5}$, β=1.5, $\xi_{i}=0.2$ for i=1,2,3,4 and implement the proposed control protocol (7). Through the analysis, all conditions (9) of Theorem 1 are satisfied*.

**Figure 2:**
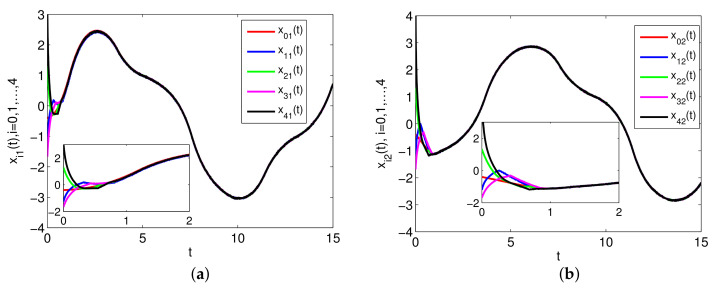
The states of $x_{i}\left(t\right),\;i=0,1,\ldots ,4$. (**a**) The states of $-x_{i1}\left(t\right)+2z\left(x_{i1}\left(t\right)\right)-1.2z\left(x_{i2}\left(t\right)\right)$; (**b**) The states of $-x_{i2}\left(t\right)+1.2z\left(x_{i1}\left(t\right)\right)+2z\left(x_{i2}\left(t\right)\right)$.

**Figure 3:**
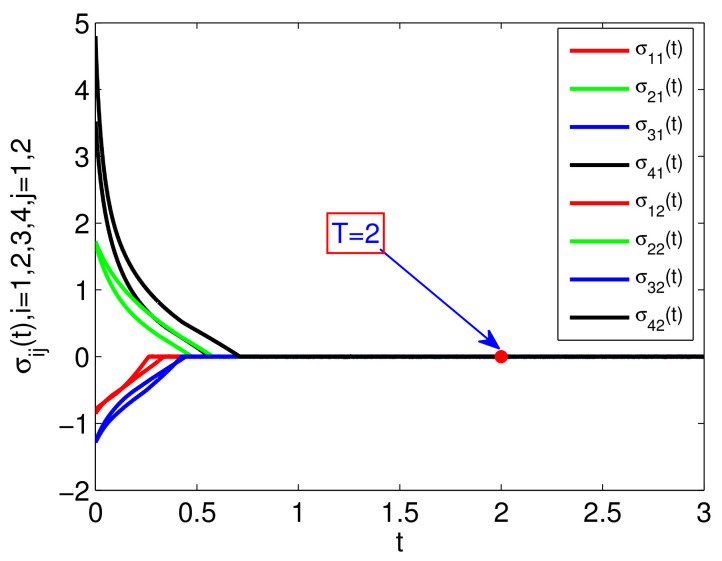
The state of σ(t).

**Figure 4:**
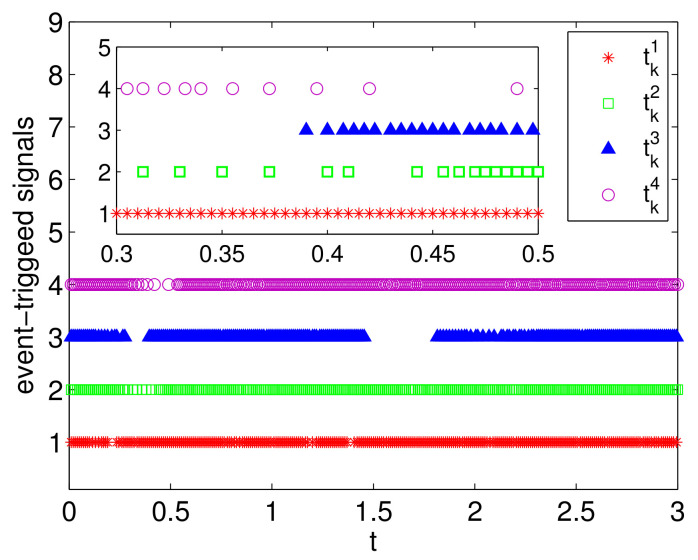
The event-triggered instants.

The simulation results are presented in Figure 2, Figure 3 and Figure 4. Specifically, Figure 2 depicts the states of all followers and the leader. It can be seen that all followers can track the leader’s state in fixed-time under the proposed sliding mode control protocol (7) and the setting time is $\tilde{T}\leq 13.86$. Based on analysis of Theorem 1, the sliding mode variable σ(t) converges to zero in fixed-time, and the setting time is T≤2, which is verified in Figure 3. The event-triggered instants under the triggering mechanism (10) are shown in Figure 4. It is shown that the event-triggered instants of each agent are different. Therefore, the results of Theorem 1 are feasible and the proposed sliding mode control protocol (7) can effectively suppress the external disturbances and realize leader-following consensus in fixed-time.

**Example 2.**  *Consider the FONMAS (24) with two leaders and four followers. The directed communication topology between two leaders and all followers are given in Figure 1b. The nonlinear function is defined as follows*

$$\begin{array}{c} f\left(x_{i}\left(t\right)\right)=\left(\begin{array}{c} -x_{i1}\left(t\right)+2z\left(x_{i1}\left(t\right)\right)-1.2z\left(x_{i2}\left(t\right)\right)\\ -x_{i2}\left(t\right)+1.2z\left(x_{i1}\left(t\right)\right)+2z\left(x_{i2}\left(t\right)\right) \end{array}\right),\;\;i=1,2,\ldots ,6. \end{array}$$

*where $z\left(x_{ij}\left(t\right)\right)=0.5(|x_{ij}\left(t\right)+1|-|x_{ij}\left(t\right)-1\left|\right)+0.01\mathrm{sgn}\left(x_{ij}\left(t\right)\right),i=1,2,\ldots ,6,j=1,2.$ The external disturbances are defined as $w_{11}\left(t\right)=w_{12}\left(t\right)=0.05\sin\left(t\right)+0.1\cos\left(t\right)$, $w_{21}\left(t\right)=w_{22}\left(t\right)=0.05\sin\left(t\right)+0.1\cos\left(t\right)$, $w_{31}\left(t\right)=w_{32}\left(t\right)=0.05\sin\left(t\right)$, $w_{41}\left(t\right)=w_{42}\left(t\right)=0.1\cos\left(t\right)$, $w_{51}\left(t\right)=w_{52}\left(t\right)=0.03\sin\left(t\right)+0.2\cos\left(t\right)$, and $w_{61}\left(t\right)=w_{62}\left(t\right)=0.06\sin\left(t\right)$. It has $∥w_{i}{\left(t\right)∥}_{\infty }\leq 0.2,i=1,2,3,4$, $∥w_{j}{\left(t\right)∥}_{\infty }\leq 0.2,j=5,6$. The control input are $u_{j1}\left(t\right)=u_{j2}\left(t\right)=0.1\sin\left(t\right)+0.1\cos\left(t\right)$, j=5,6. We choose the controller parameters $K_{1}=1.7$, $K_{2}=2$, $K_{3}=1.5$, $K_{4}=24.5$, $\eta =\frac{7}{5}$, β=1.2, ξ=1 and implement the proposed control protocol (30). Through simple calculation, we can verify that all conditions of Theorem 3 are satisfied*.

**Figure 5:**
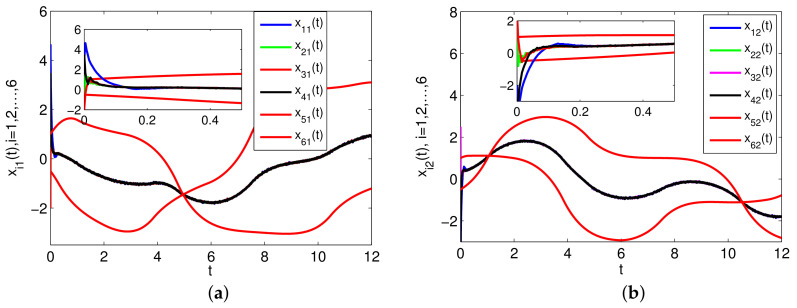
The states of $x_{i}\left(t\right),\;\;i=1,2,\ldots ,6$. (**a**) The states of $-x_{i1}\left(t\right)+2z\left(x_{i1}\left(t\right)\right)-1.2z\left(x_{i2}\left(t\right)\right)$; (**b**) The states of $-x_{i2}\left(t\right)+1.2z\left(x_{i1}\left(t\right)\right)+2z\left(x_{i2}\left(t\right)\right)$.

**Figure 6:**
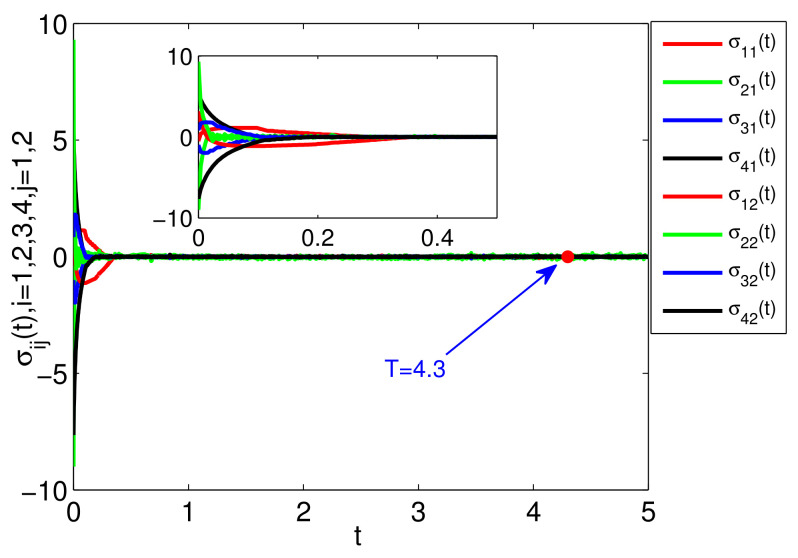
The state of σ(t).

**Figure 7:**
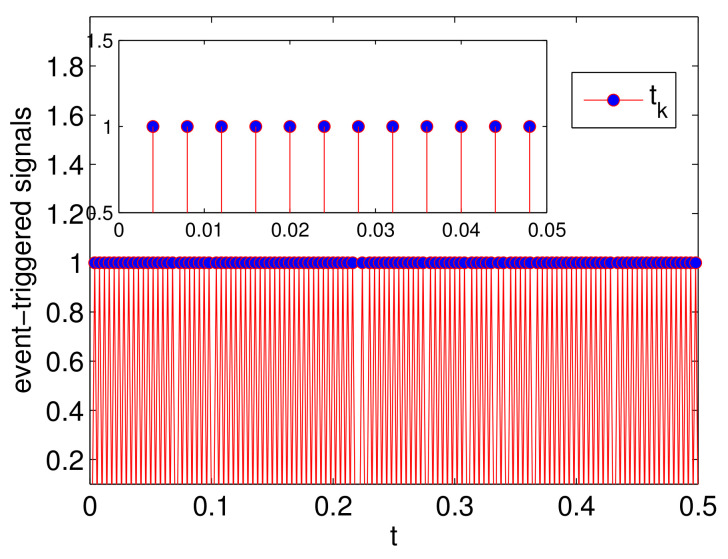
The event-triggered instants.

The simulation results are presented in Figure 5, Figure 6 and Figure 7. Specifically, Figure 5 shows the states of four followers and two leaders. It can be seen that all followers’ states gradually achieve consensus and fall into the convex hull of the leaders’ states in fixed-time and the setting time is $\hat{T}\leq 11.3$. Figure 6 shows the evolution of sliding mode variable σ(t). The sliding mode surface can be reached in fixed-time, and the settling time is $\bar{T}\leq 4.3$. The triggering interval under the event-triggered mechanism (33) is presented in Figure 7. In order to show the event-triggered intervals more clearly, we only give the simulation result for a short period of time, from which we can see that the Zeno phenomenon can be excluded. Different from the distributed event triggering condition (10), we employ a centralized trigger function, which also can ensure the reachability of the consensus. In particular, if the FONMAS (24) with one leader, the containment control can be reduced into leader-following tracking problem.

## 5. Conclusions

In this paper, considering external disturbances, the leader-following consensus and containment control of FONMASs are studied. Two kinds of event-triggered integral SMC protocols are designed, which can well suppress the external disturbances and make the FONMASs achieve consensus in fixed-time. Based on fixed-time stability theory and inequality technique, some criteria are obtained and the Zeno behavior can be avoided. The effectiveness of the proposed protocols are verified by several numerical simulations. In the future work, the consensus of higher-order MASs with dynamic event-triggered communication mechanism will be considered.

## Data Availability

Not applicable.

## Abbreviations

The following abbreviations are used in this manuscript:

MASs	Multi-agent systems
FONMASs	First-order nonlinear multi-agent systems
SMC	Sliding mode control
